# Insect Larvae Meal (*Hermetia illucens*) as a Sustainable Protein Source of Canine Food and Its Impacts on Nutrient Digestibility and Fecal Quality

**DOI:** 10.3390/ani11092525

**Published:** 2021-08-27

**Authors:** Amr Abd El-Wahab, Laura Meyer, Mareike Kölln, Bussarakam Chuppava, Volker Wilke, Christian Visscher, Josef Kamphues

**Affiliations:** 1 Department of Nutrition and Nutritional Deficiency Diseases, Faculty of Veterinary Medicine, Mansoura University, Mansoura 35516, Egypt; amrwahab5@mans.edu.eg; 2 Institute for Animal Nutrition, University of Veterinary Medicine Hannover, Foundation, Bischofsholer Damm 15, D-30173 Hannover, Germany; sekretariat-tierernaehrung@tiho-hannover.de (L.M.); service-tierernaehrung@tiho-hannover.de (M.K.); bussarakam.chuppava@tiho-hannover.de (B.C.); volker.wilke@tiho-hannover.de (V.W.); josef.kamphues@tiho-hannover.de (J.K.)

**Keywords:** dog food, black soldier fly larvae, nutrient utilization, fecal characteristics

## Abstract

**Simple Summary:**

Insects are considered an optimistic component for animal diets as an alternative to some of the common feedstuffs. Therefore, the present research studied the effect of including black soldier fly larvae meal in a canine diet on nutrient digestibility and fecal quality compared to that of poultry meal. Within this study, it was possible to include the insect larvae meal to replace 30% of dry matter of the basic extruded diet. The research indicated that insect larvae meal showed the highest apparent digestibility for protein and fat without any negative effects on fecal scores, stating it as a potential alternative food ingredient for dogs. These findings suggest that insect larvae meal can be considered a suitable applicable ingredient for canine food that might potentially be included in pet food formulations.

**Abstract:**

Insect larvae meal has been proposed as a sustainable protein source for animal diets. This study aimed to provide information on including black soldier fly larvae meal (BSFL; *Hermetia illucens*) in comparison to poultry meal (PM) in the canine diet with regard to digestibility and fecal characteristics. In light of this trend, the levels of PM or BSFL meal were added to replace about 30% of dry matter of the basic extruded diet. Six Beagle dogs (BW 9.64 kg) were included in a cross-over experiment. Dogs fed a BSFL meal-based diet showed higher (*p* < 0.05) apparent protein digestibility (82.3%) compared to those offered a PM-based diet (80.5%). Apparent digestibility for fat was higher (*p* < 0.05) in groups fed the BSFL meal-based diet (94.5%) compared to those offered the PM-based diet (91.6%). The fecal consistency scores for dogs fed both diets were within an acceptable range (well-formed and firm). Fecal dry matter content was higher (*p* < 0.05) for dogs fed the PM-based diet (33.0%) compared to those offered the BSFL meal-based diet (28.0%). Including BSFL meal in dog food can be an appropriate source of protein without any negative effects on nutrient digestibility and fecal quality.

## 1. Introduction

In the coming 30 years, the global population is estimated to reach 10 billion [1]. By then, global food production will need to produce sufficient food and nutrients for the increased population and also address the environmental impacts of food production [2]. Thus, this situation leads to exploring alternative, sustainable protein sources in a global aspect. Insects have received considerable attention as a sustainable, natural, and novel protein source for humans and pet animals [3]. The acceptance of insects as food for pet animals is primarily driven by personal attitudes towards food (i.e., acceptance of novel foods), cultural exposure, interest in the environmental impact, and concerns about food sustainability [4]. Insects for pet food application could serve as a sustainable protein source [5]. It is worth considering the environmental and economic benefits of this new trend of the insect industry. Insects have the ability to provide a high nutritive value with lower environmental impacts (environmentally friendly) compared to that of conventional livestock sector [6]. Insects require fewer resources and emit fewer greenhouse gas emissions compared with those of livestock raised for food production [7]. Moreover, livestock is considered an important contributory factor to climate change. The 20 billion domesticated food-producing animals produce between 5.6 and 7.5 Gt CO_2_ equivalents/year, with cattle being responsible for 64–78% of these emissions [8]. However, it was estimated that greenhouse gas emissions are much lower for insects (2–122 g/kg mass gain) than for pigs and beef cattle (80–1130 and 2850 g/kg mass gain, respectively) [9]. Production of one kilogram of black soldier fly larvae (BSFL; *Hermetia illucens* (L.), Diptera: Stratiomyidae) protein is estimated to generate three kilograms of CO_2_ equivalents when BSFL are fed a feed-grade substrate and approximately 19 kg when fed a food-grade substrate [10]. Moreover, based on life cycle assessment, insect protein may have lower environmental influence (e.g., lower land use, lower water use, less emission of CO_2_ equivalents) than animal protein from other sources [11]. Additionally, insects could also be grown on food waste, contributing to circular economies [12]. Therefore, insects are considered a more environmentally friendly source of animal protein than poultry, pig, and beef cattle. BSFL have received great attention for their ability to convert organic waste into high-value biomass [13]. BSFL are able to feed on a variety of organic materials such as cow manure, fish offal, brewery by-products, restaurant waste, and sewage sludge [14].

Many researchers have studied the differences in insect nutritional content [15]. Their nutritional composition can vary depending on the rearing substrate, but in general, BSFL contain around 400 g of crude protein per kilogram of dry matter (DM) and 300 g crude fat per kilogram of DM [16]. According to Heide [17], the crude protein and crude fat contents of the defatted BSFL meal were about 675 g/kg DM and 62 g/kg DM, respectively. Insect protein-based nutrition has been declared as a high-quality and efficient food source [6]. The Food and Agricultural Organization of the United Nations stressed the significance of insect protein as a possible future nutrition and animal food source [11]. Due to the nutritional composition of insects, BSFL have been used in parts of the world as an ingredient in feed for various animals such as poultry and fish [18]. Compared with crickets and mealworms, BSF boasts a more stable nitrogen and phosphorus composition and has a more advantageous feed conversion ratio, besides its immediate potential for large-scale production [19]. Dry diets containing BSFL meal were preferred by dogs over those containing yellow mealworm (*Tenebrio molitor*) meal [20,21]. Dogs have been proven to eat dry diets containing 5, 10, or 20% BSFL meal according to Yamka et al. [22]. Although not all data comes from peer-reviewed studies, specific information on the nutrient digestibility of insects in dogs has just been published. The average apparent fecal protein digestibility of commercial dog food is close to 80% [23], implying that insect meals are comparable to other protein sources.

Owners are in intimate contact with their pets’ feces and may link fecal characteristics (volume, consistency, odor, color, and frequency of defecation) to intestinal health and food nutritional quality [6]. Extruded diets with increasing quantities of banded cricket meal (8%, 16%, or 24%) resulted in well-formed feces in dogs [5]. Though there are few studies, it appears that incorporating insect meals in dry extruded pet diets does not interfere with intestinal function and results in satisfactory fecal consistency.

Finding an alternative and sustainable protein source that is nutritionally adequate and safe for pets is essential for future food security [24]. Additionally, the palatability of a novel protein source and its effects on health status and fecal quality are considered the key attribute to the success of a canine food [3]. Against this background, the present study aimed to provide information on the possibility of including BSFL meal as an insect protein source in canine food regarding nutrient digestibility and fecal characteristics compared to a poultry meal (PM) as a common animal protein source.

## 2. Materials and Methods

The Animal Welfare Officer of the University of Veterinary Medicine Hannover, Foundation, Germany gave his approval to this study design.

### 2.1. Experimental Design

The digestibility study at the Institute for Animal Nutrition, University of Veterinary Medicine Hannover, Foundation, Germany included six healthy female Beagle dogs. The dogs had a median age of three years and a mean body weight (BW) of 9.64 ± 0.682 kg at the start of the trial (range 12–48 months). The body condition score during the whole experimental trial was 4.98 ± 0.312 out of 9 in accordance with Laflamme [25]. The health status of the dogs was checked before the beginning of the experiment by physical examination, and they were vaccinated and dewormed. The dogs lived in 3.35 × 2.80 m kennels with daily access to an outside playground for exercise and socialization, where they were acclimatized to the experimental diets. During the digestibility tests, the dogs were housed individually in 4.00 × 2.05 m kennels to enable fecal collection. The trial was conducted in a cross-over model. During this study, each dog was assigned once to PM- or BSFL meal-based diets. The animals were adapted to the diet for five days; followed by five days of fecal collection for individual estimation of the apparent nutrient digestibility and fecal scores. The amount of food offered was calculated by a formula according to their energy requirements (0.5 MJ metabolizable energy × BW^0.75^/d) based on the energy requirement prediction equation for maintenance of adult dogs [26]. The food offered was adjusted weekly to keep the animals’ BW constant. The animals were fed once per day and received water ad libitum. The amount of food offered and refused was recorded at each meal to calculate food preference. Every day, a metal container was weighed and filled with new water. The remaining amount of water was weighed after each day of the trial to calculate the daily water intake.

### 2.2. Diets Production

**Figure 1:**
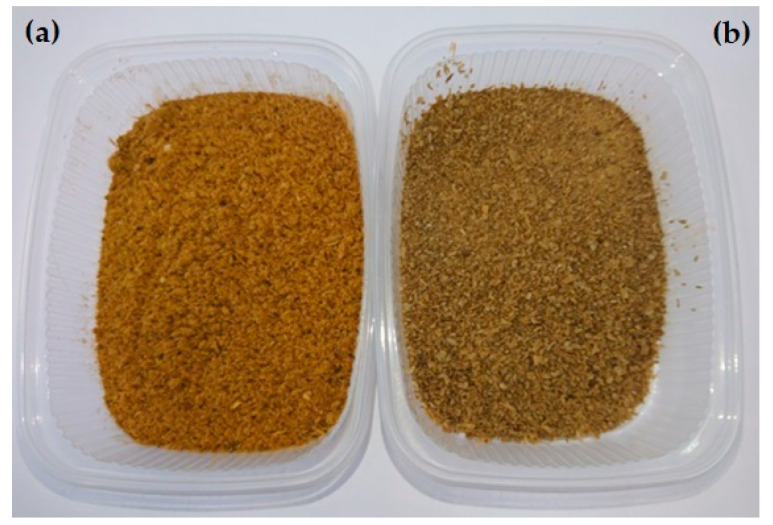
Experimental ingredients used in the canine food. (**a**) Poultry meal; (**b**) insect larvae meal (Photo ©L. Meyer/TiHo).

The two experimental diets were obtained by replacing about 30% DM of the basic diet with either PM or BSFL meal (Figure 1). Both experimental diets were complete foods, however, the ingredients (PM or BSFL meal) were mixed with the basic diet daily during each mealtime. The PM contained a processed poultry protein of only ground and rendered parts from slaughtered poultry (as undeveloped eggs and intestines), whereas the BSFL meal contained larvae of *Hermetia illucens*, which was freeze-dried, ground, and partially defatted to 195 g crude fat/kg DM.

An extruded basic diet (Jonker Petfood B.V., Waalwijk, The Netherlands) contained wheat, wheat gluten, rice protein, broken rice, linseed, sugar beet pulp, brewer’s yeast, palatability enhancer, dicalcium phosphate (Table 1). The basic diet was manufactured by extrusion technologies using a temperature of 107 °C and a pressure of 30 bar for 1 min. The diet was pressed to 8 mm in diameter. Subsequently, the diet was dried at 120 °C for 30 min, vacuum coated, cooled, and packaged.

### 2.3. Chemical Analysis

The Association of German Agricultural Analytic and Research Institutes (VDLUFA) methodologies were used to determine the nutrients in the diets and feces samples [27]. Weighing the samples before and after drying them at 103 °C was used to calculate the DM concentration. Weighing the samples before and after combustion at 600 °C was used to detect the crude ash content in the muffle furnace. The total N content was also measured using the Dumas incineration method (Vario Max CNS, Elementar Analysensysteme GmbH, Langenfeld, Germany). The crude fat content was determined after acid digestion in Soxhlet equipment. The content of crude fiber was estimated after washing in diluted acidic and alkalic solutions and subsequent drying at 103 °C (Fibertec^TM^ 2010, Foss Innovation Centre, Hillerød, Denmark). The starch content of the diets was determined using a polarimetrical technique (Unipol 2020, Schmidt + Haensch GmbH & Co., Berlin, Germany). Sugar in the samples was analyzed by using the Luff-Schoorl method after microwave incineration (Ethos lab, MLS GmbH, Leutkirch, Germany). The calcium content was determined by atomic absorption spectrometry (Solaar AA Spectrometer M Series, Thermo Fisher Scientific, Inc., Waltham, MA, USA) in accordance with Slavin [28]. A photometric characterization of phosphorus content was based on the vanadate molybdate method in accordance with Gerickend and Kurmies [29]. Ion-exchange chromatography (AA analyzer LC 3000, Eppendorf-Netheler-Hinz GmbH, Maintal, Germany) was used to analyze amino acid contents. The content of nitrogen free extract was calculated. The chemical composition of the diets was used to determine the level of metabolizable energy in accordance with the National Research Council (NRC) [30].

### 2.4. Chemical Composition of Experimental Diets

Chemical analysis of the basic diet is shown in Table 2. The contents of crude protein, crude fat, and crude fiber were about 222, 106, and 16.5 g/kg DM, respectively.

The chemical composition of the experimental diets in this study varied considerably due to different ingredient profiles (Table 3 and Table 4).

The moisture content between the experimental canine foods was nearly similar (range: 913–929 g/kg, Table 5). The crude ash and crude protein contents were 82.5 and 375 g/kg DM in the PM-based diet, while their levels were about 58.9 and 326 g/kg DM in the BSFL meal-based diet, respectively. The contents of crude fat and crude fiber were about 133 and 39.5 g/kg DM, respectively, in the BSFL meal-based diet. Meanwhile, the PM-based diet had about 99.3 and 14.7 g/kg DM for crude fat and crude fiber contents, respectively. The analyzed contents of some amino acids slightly differed between both experimental diets. The metabolizable energy was comparable between both diets (1.54–1.65 MJ/100 g as fed).

### 2.5. Food Intake Scoring and Apparent Digestibility

In accordance with Zahn [31], the spontaneous acceptance “food intake scoring” (palatability and the speed of food intake) was divided into three grades (1 = lowest acceptance; 2 = moderate acceptance; 3 = highest acceptance).

The total fecal collection method was used to perform the apparent nutrient digestibility [32], consisting of an initial phase of five days of adaptation to the diet, followed by five days of fecal collection. During the collection period, fresh feces were collected daily from the concrete floor. After being weighed, in a subsample of 10% of the fresh feces per animal and day, the DM content was determined. Thereafter, the remaining fecal samples were stored at −20 °C. At the end of the trial, five-day fecal samples from each dog were thawed, mixed, and homogenized. The apparent digestibility was calculated using the following formula [26]:

Apparent digestibility (%) = ((food − excreta)/food) × 100

### 2.6. Fecal Quality

Every day, the number of defecations was counted. Fecal scores were recorded using a 5-point scale (1 = very hard; 2 = solid, well-formed, “optimum”; 3 = soft, still formed; 4 = pasty, slushy, and 5 = watery diarrhea) according to Moxham [33]. The photo of fecal scoring was shown in a previous study [34]. The feces shaping scores were determined in accordance with Zieger [35], using a 5-point scale (1 = individual feces mass; 2 = shaped, with strong constrictions at the fecal surface, “optimum”; 3 = shaped with fissures at the fecal surface; 4 = pasty, slushy, and 5 = shapeless).

### 2.7. Statistical Analysis

The statistical analysis was carried out using SAS^®^ Enterprise Guide^®^, version 9.3 of the Statistical Analysis System for Windows (SAS Institute, Inc., Cary, NC, USA). Mean values as well as the standard deviation (SD) of the mean were calculated for all parameters. All measured or recorded parameters were separately analyzed and served as the basis for the calculation. First, the data were checked for normal distribution and then further tested with the *t*-test as well as Wilcoxon test. The significance level was determined at *p* < 0.05.

## 3. Results

Food intake scoring was similar among dietary treatments, and no refusals were observed throughout the duration of the trial. The daily food intake was comparable between both groups (161 and 164 g/dog/DM for groups fed PM- and BSFL meal-based diets, respectively). The slight difference in food intake due to the individual needs of the dogs were determined in the context of BW development and the respective body condition score. Additionally, the water intake was comparable between both groups (508 mL/d and 515 mL/d for groups fed PM- and BSFL meal-based diets, respectively). The BW of the dogs did not change during the study. Body weight was similar among groups at the start of the trial (*p* > 0.05) and remained constant throughout the trial.

### 3.1. Apparent Nutrient Digestibility

The apparent digestibility of organic matter in groups fed either the PM-based diet or BSFL meal-based diet was similar (83.6%), as shown in Table 6. Apparent protein digestibility was significantly higher for dogs fed the BSFL meal-based diet compared to those fed the PM-based diet (82.3% vs. 80.5%). Additionally, crude fat digestibility was higher (*p* < 0.05) for dogs fed the BSFL meal-based diet (94.5%) compared to that of those offered the PM-based diet (91.6%). Nonetheless, nitrogen free extract digestibility was comparable for dogs fed the PM-based diet and the BSFL meal-based diet (88.1% and 88.9%, respectively).

### 3.2. Fecal Quality

Defecation frequency (average of 2.07 and 2.40 per day for dogs offered the PM-based diet and the BSFL meal-based diet, respectively) was not affected by the type of protein ingredient added to the diet (Table 7). The scores of fecal consistency and shaping were very close to the desired optimal score (score 2) in both groups (Table 7). However, fecal consistency and shaping scores were higher (*p* < 0.05) for dogs fed the PM-based diet in comparison to those of dogs offered the BSFL meal-based diet. The mass of wet feces was significantly higher for dogs fed the BSFL meal-based diet (569 g fresh feces/5 d) compared to that of those offered the PM-based diet (492 g fresh feces/5 d). The fecal DM content differed significantly between both groups (33.0% vs. 28.0% for groups fed the PM-based diet and the BSFL meal-based diet, respectively).

## 4. Discussion

Insects are currently considered to be one of a number of alternative and sustainable protein sources for pets [3]. In the present study, the effects of protein source (BSFL meal) in canine food were investigated regarding apparent nutrient digestibility and fecal characteristics.

In the current study, it was observed that food intake scoring was similar among dietary treatments, and no refusals occurred. It was proven that the smell of food also plays a crucial role in indicating nutritional preferences [36]. Moreover, in the available literature, there is no information about the inclusion of insect species in companion animal diets as an aroma additive and consequently, the palatability. However, Kierończyk et al. [37] suggested that insects such as BSFL may be attractive to dogs. Thus, the possibility of insect application to dog diets provides the double benefit of an encouraging palatability/aroma and consequently, the food intake as well as an additional high-quality nutrient source.

### 4.1. Nutrient Digestibility

Organic matter digestibility in our study was similar for dogs offered either PM- or BSFL meal-based diets (83.6%). A similar result was obtained by Penazzi et al. [38], where organic matter digestibility was similar in dogs fed the control (processed deer protein source up to 40% as fed) and BSFL meal (36.5% as fed). In vitro organic matter digestibility of house crickets was reported to be 88% as observed by Bosch et al. [6], which was similar to that of PM (85.8%). One factor that could affect organic matter digestibility in pets is the content of crude fiber and its source (soluble or insoluble) as described by Zentek [39] and De Godoy et al. [40]. Previously, apparent organic matter digestibility of commercial pet diets was negatively linked with fiber content [41]. It is well-known that increasing dietary fiber content was associated with reduced organic matter digestibility in pets [42]. Meyer and Zentek [43] and Monti et al. [44] stated that the increase in crude fiber by 1% in the DM of the canine food was accompanied by 1.6% decreased organic matter digestibility due to lower microbial decomposition in the colon as a result of accelerated food passage. In the present study, however, dietary fiber content in the BSFL meal-based diet was higher (39.5 g/kg DM) compared to that of the PM-based diet (14.7 g/kg DM), but with no effect on organic matter digestibility. Chitin, a component of the insect exoskeleton that is recovered in fiber analyses [45] and that monogastric animals are unable to digest [5,46], may explain the increased fiber content of insect-based diets. Chitin has previously been linked to a reduction in insect digestibility in livestock and aquaculture [47]. However, in the present study, crude fiber content did not seem to dictate apparent organic matter digestibility, implying that our findings may not be biologically significant, or that all diets were below the physiological maximum for fiber.

Protein concentration varies from 18% to 40% in dietary formulas for healthy adult dogs [48]. High-protein diets may result in greater amounts of undigested protein reaching the colon compared with those of low-protein diets [48] and consequently affect the digestibility. However, if the aim is to decrease fermentation in the hindgut, the choice of protein source used in the dietary formula is of greater importance than protein concentration [48]. In the current study, the apparent protein digestibility in both diets ranged between 80.5 and 82.3%. Our results are consistent with previous studies regarding the levels of apparent protein digestibility. According to the European Pet Food Industry Federation [49], the protein minimum requirement was based on an apparent protein digestibility of 80%, which roughly conforms to our findings. Apparent protein digestibility of canine foods containing BSFL ranged from 73.2 to 87.2%. The typical apparent fecal protein digestibility of conventional dog food is around 80% [23,50], implying that insect meals are comparable to traditional protein sources. A similar result was obtained by Penazzi et al. [38], where apparent protein digestibility was higher in dogs fed the control (processed deer protein source) and BSFL meal. Similarly, Lei et al. [51] observed that increasing levels of BSFL meal inclusion (at 0, 1, and 2%) in Beagle dog diets raised protein digestibility compared to that of the control diet. As pointed out by Penazzi et al. [38], compared to that of vertebrate protein meal, collagen is probably limited in insect meal. This could also explain the higher level of protein digestibility of the BSFL meal-based diet compared with that of the PM-based diet.

Additionally, one factor that could affect protein digestibility is dietary crude ash content. In our study, the crude ash content in the PM (as ingredient) was about 147 g/kg DM vs. 68.2 g/kg DM for the BSFL meal (as ingredient). Consequently, the extruded complete foods had differences in crude ash content (82.5–58.9 g/kg DM for PM- and BSFL meal-based diets, respectively), with an influence on protein digestibility. Similarly, according to Meyer and Mundt [52], higher crude ash content in food possibly leads to insufficient acidification of the chyme, which may result in lower protein digestibility. Siebert [53] stated that incomplete dissolution of minerals from connective tissue of bone meal resulted in impeded proteolysis, and high crude ash contents in PM are likely to originate from bone fractions, for example. The high ash content of some animal by-product meals negatively affects the quality of their protein, as essential amino acid levels per unit of protein are reduced [54], limiting their inclusion in diet formulations. Penazzi et al. [38] speculated that the control diet (processed deer protein source) had a decreased crude protein digestibility compared to that of BSFL meal-based diet in dogs due to the higher crude ash content. Therefore, the low crude ash content of a BSFL meal-based diet represents an advantage over other PM-based diets, which generally have a high mineral content.

Another factor that could influence protein digestibility is dietary fiber content [55]. In the current study, although crude fiber content in the BSFL meal-based diet was two times more than the level in the PM-based diet (39.5 vs. 14.7 g/kg DM), this had no effect on protein digestibility. The various effects of fiber on digestibility in pets, according to De Godoy et al. [40], are likely to alter the consequences of fiber levels, type (amount of fermentability), and the dietary matrix. Protein digestion in dogs was not affected by changing the source (beet pulp and maize fibers) or concentration (total dietary fiber 8.40–10.2%) [56]. In contrast to our results, Siebert [53] found negative effects of high fiber content in canine food on protein digestibility when adding lignocellulose. Digestibility of crude protein decreased as total dietary fiber consumption increased in dogs [57]. The microbiome can both trap nitrogen as bacterial protein and liberate nitrogen as ammonia, therefore, fermentable carbohydrates may influence protein digestibility through lower tract metabolism [55]. Thus, in our study, certain effects on protein digestibility due to crude fiber content can be excluded or rather neglected because of the low contents and/or differences. Overall, PM as an ingredient may present variable amounts of low bioavailable materials, such as residual bone, feathers, feet, and beaks, and can be produced under variable processing conditions [54], creating variability in the composition and digestibility of the ingredient.

The type of diet (BSFL meal-based diet) used in the canine food in the present study had a significant positive effect on apparent fat digestibility, which could be related to some factors. One of these factors is dietary fat content. In the current study, dietary fat content of the BSFL meal-based diet was about (133 g/kg DM) with a fat digestibility of about 94.5%, while the dietary fat content of the PM-based diet was about (99.3 g/kg DM) with a fat digestibility of about 91.6%. Zuo et al. [58] found that the fat digestibility increased to about 97% when the amount of dietary fat increased. Hill et al. [59] noted that the digestibility of fat reached about 99% when the dogs ate diets containing a high amount of fat (about 320 g/kg DM). This increase in fat digestibility is in line with the current study’s findings, which show that dietary fat content promotes fat digestibility. Thus, it can be assumed that the apparent digestibility of fat tends to increase as dietary fat increases. Furthermore, apparent fat digestibility is affected by lipid type and processing conditions [60]. Notably, the lipid type was the same for both diets (plant source; sunflower oil). However, the lipid type and/or fatty acids in the PM or BSFL meal could not be neglected and hence may have contributed to fat digestibility differences observed in the present study. It is well-known that a high content of saturated fatty acids (especially lauric acid) and monounsaturated fatty acids has been found in BSFL, while the contents of eicosapentaenoic acid (C20:5) and docosahexaenoic acid (C22:6) were low [61,62,63]. The lipids present in the PM ingredient are generally rich in monounsaturated fatty acids (particularly oleic acid) and total n-6 polyunsaturated fatty acids but are low in n-3 polyunsaturated fatty acids, eicosapentaenoic acid (C20:5), and docosahexaenoic acid (C22:6) [64,65]. Consequently, the contents of saturated fatty acids and/or total n-6 polyunsaturated fatty acids in diets of dogs could influence fat digestibility.

Another factor affecting fat digestibility in a variable way could be the crude ash content. Actually, in our study, crude ash content in the PM-based diet was higher (+23.6 g/kg DM) than that of the BSFL meal-based diet. However, fat digestibility was significantly higher for dogs offered the BSFL meal-based diet. Similarly, Meyer and Zentek [43] observed that fat digestibility decreased with an increased level of crude ash content in dog food because of a possible soap formation.

Including the BSFL meal-based diet did not affect the digestibility of the nitrogen free extract, which did not differ significantly from the PM-based diet. This fact may partially be explained by the comparable content of the nitrogen free extract in both diets (428 vs. 442 g/kg DM for PM- and BSFL meal-based diets, respectively). Moreover, the levels of starch and sugar in both diets were identical (328 and 18.4 g/kg DM, respectively).

### 4.2. Fecal Characteristics

Fecal quality is an important index in the evaluation of dog foods. There are many variables that affect fecal quality, including nutrient digestibility, fiber content, DM intake, and fat tolerance [66]. In the current study, fecal scores were maintained at acceptable levels with an average of 2.5 for each treatment. The extruded diet with BSFL meal resulted in a fecal consistency score that varied significantly closer to the optimal value (score 2) than that resulting from the PM-based diet. Thus, a clearly positive influence of BSFL meal inclusion compared to PM ingredient on fecal quality could be demonstrated. Our findings are comparable to those of Yamka et al. [22], who found that all diets containing 20% BSFL meal gave dogs an optimal fecal consistency score. A recent study found that when dogs were fed extruded meals with increasing quantities of cricket meal (8, 16, or 24%), their feces remained well-formed [5]. Though the number of studies is still limited, it seems that adding insect meals to dry extruded pet foods does not influence intestinal functioning and leads to an acceptable fecal consistency score [6]. Other factors such as dietary protein content that affect the fecal consistency score should not be neglected either. In the current study, the content of crude protein in the PM-based diet was about 49 g/kg DM higher than that found in the BSFL meal-based diet. Nery et al. [48] observed a softer fecal consistency at higher protein levels in canine food and explained this by increased fermentative degradation in the colon. Zentek et al. [67] described an influence of the amount and type of protein source on fecal quality, as the softer fecal consistency was particularly due to a higher collagen content in the protein fraction of the food. Moreover, Weber et al. [68] stated that the increase in proteolytic fermentations in the hindgut is one of the dietary factors causing greater moisture in feces (and negatively influencing fecal quality).

In the current study, dogs fed the BSFL meal-based diet had a 77 g higher wetter fecal output for five days than that of the dogs fed the PM-based diet. The amount of fecal output may be influenced by food intake, nutrient digestibility, chemical composition of the diet, and physiological state of the animal [69]. Although the water-holding capacity of the dietary ingredients is a factor, greater nutrient digestibility usually results in lower fecal output [69], even though the increased fecal output may be explained by an increase in dietary fiber [70]. Previous studies have shown an increase in wet fecal weight with the increase in dietary fiber [71]. From another point of view, Jarett et al. [72] found that diets containing crickets supported the same level of gut microbiome diversity in dogs as a standard healthy balanced diet. This suggests that the increase in wet fecal output associated with higher BSFL meal inclusion was not related to microbial abundance. The DM fecal output is usually unaffected by an increase in wet fecal output, implying that the main contributor is increased fecal water content. Similarly, in this study, the DM fecal output was also not significantly influenced.

Notably, in the current study, the fecal DM content remained significantly low at 28.0% when using BSFL meal in about 30% DM of the basic diet. Many different factors may markedly affect the fecal DM content in pets. Protein digestion and absorption are considered to be one of the dietary factors affecting fecal DM content [68]. If protein is present but not absorbed, the dietary amino acids in that protein are unavailable to the host and serve as a nitrogen source for proteolytic bacteria, resulting in low fecal quality [73]. Another factor is fiber fermentation activity. High positive correlations were found in dogs between fermentation activity on the one hand and moisture content of feces on the other hand [39,71,74]. This conclusion could be related to fiber’s “bulking impact”, and it appears to be most strongly linked to insoluble fiber sources that are both poorly fermentable and have high water-binding capacity [74]. Soluble fiber typically has an increased extent of fermentation by gastrointestinal microbes, yielding short-chain fatty acids (mainly acetate, propionate, and butyrate). Short-chain fatty acids play a variety of physiological roles, including increased water absorption in the gastrointestinal tract [75]. However, overdosing of butyrate might induce an osmotic effect, resulting in increased fecal moisture content and worse fecal consistency [75]. Further research is needed to access the water-binding capacity of chitin. Based on the current knowledge, very few studies are available in the literature on the chitin content of BSFL meal. Kroeckel et al. [76] reported a chitin amount of 96 g/kg DM in defatted BSFL. However, Schiavone et al. [77] revealed a relevant low chitin content (50 and 69 g/kg DM for partially defatted BSFL meal and highly defatted BSFL meal, respectively). Overall, there are contradictory statements regarding the relationship between fecal DM content and fecal consistency score. In the present study, there was no correlation between these two parameters. According to some authors, however, the correlation is given [35,43,78], but in other studies, this is not comprehensible [39,79]. Heide [17] did not show a significant difference in the DM content of feces, but fecal consistency was found to be significantly firmer when the diet without insect meal was used. Beloshapka et al. [80] suggested that significant differences in fecal DM content could be achieved when different amounts of a specially processed soy protein were added, but not in terms of fecal consistency.

## 5. Conclusions

In conclusion, the diets tested in this study were well-accepted, and the dogs remained healthy throughout the study. The present study suggests that including BSFL in extruded diets for dogs offers a promising alternative source of dietary protein, particularly in relation to the digestibility profile and fecal quality. Overall, the high spontaneous acceptance of tested foods as well as the positive influence of insect meal on nutrient apparent digestibility and fecal quality deserve a special mention. Our findings further indicate the need for more research into the bioavailability of amino acids and the health status of dogs as a result of longer-term feeding of insect meal-containing diets, particularly with regard to food allergy.

## Figures and Tables

**Table 1:** Ingredient composition of the basic diet.

Ingredient, % as Is Basis	Basic Diet
Wheat	29.1
Broken rice	29.1
Wheat gluten	8.81
Rice protein	8.81
Sunflower oil	6.84
Palatability enhancer	3.00
Sugar beet pulp	3.00
Brewer’s yeast	2.00
Linseed	2.00
Dicalcium phosphate	3.24
Minor components	4.10

**Table 2:** Chemical analysis of the basic diet.

Parameter	Unit	Basic Diet
Dry matter	g/kg as is basis	915
Crude ash	g/kg DM	54.9
Crude protein		222
Crude fat		106
Crude fiber		16.5
Nitrogen free extract		600
Calcium		9.92
Phosphorus		3.86
Metabolizable energy ^1^	MJ/100 g as is basis	1.54

^1^ Metabolizable energy content of the diets was done in accordance with NRC [30].

**Table 3:** Chemical composition of the experimental raw ingredients.

Parameter	Unit	PM	BSFL Meal
Dry matter	g/kg as is basis	962	909
Crude ash	g/kg DM	147	68.2
Crude protein		733	570
Crude fat		83.7	195
Crude fiber		10.4	93.0
Nitrogen free extract		26.5	74.3
Starch ^1^		n.d.^1^	n.d.^1^
Sugar ^1^		n.d.^1^	n.d.^1^
Calcium		35.2	11.9
Magnesium		1.40	2.56
Phosphorus		23.0	8.36
Sodium		5.88	0.890
Potassium		10.0	11.2
Cholride		21.5	3.18
Sulfur		8.98	4.74
Copper	mg/kg DM	11.6	11.7
Zinc		112	116
Iron		339	448
Manganese		24.8	230

^1^ n.d. = not determined. PM = poultry meal, BSFL = black soldier fly larvae, DM = dry matter.

**Table 4:** Levels of amino acids in the experimental raw ingredients (g/kg DM).

Amino Acid	PM	BSFL Meal
Aspargine	63.7	60.4
Threonine	26.1	23.8
Serine	29.7	26.2
Glutamine	96.2	65.9
Glycine	74.9	31.4
Alanine	53.3	41.3
Valaline	35.3	36.9
Cysteine	7.09	4.65
Methionine	12.8	9.31
Ileucine	28.8	27.4
Leucine	50.6	43.2
Tyrosine	22.8	38.6
Phenylalanine	27.5	28.3
Histadine	14.8	16.5
Lysine	47.1	39.2
Arganine	50.3	30.9
Proline	47.8	33.6

PM = poultry meal, BSFL = black soldier fly larvae.

**Table 5:** Chemical composition of the experimental diets.

Parameter	Unit	PM-Based Diet	BSFL Meal-Based Diet
Dry matter	g/kg as is basis	929	913
Crude ash	g/kg DM	82.5	58.9
Crude protein		375	326
Crude fat		99.3	133
Crude fiber		14.7	39.5
Nitrogen free extract		428	442
Starch ^1^		328	328
Sugar ^1^		18.4	18.4
Calcium		17.5	10.5
Phosphorus		9.60	5.21
Lysine		21.1	18.8
Methionine		8.13	7.08
Threonine		12.3	11.6
Metabolizable energy ^2^	MJ/100 g as is basis	1.54	1.65

^1^ only determined in basic diet; ^2^ calculated according to NRC [30].

**Table 6:** Apparent nutrient digestibility (%) of dogs fed the poultry meal- and insect meal-based diets (mean ± SD).

Parameter	PM-Based Diet	BSFL Meal-Based Diet
Organic matter	83.6 ± 0.38	83.6 ± 0.21
Crude protein	80.5 ^b^ ± 1.07	82.3 ^a^ ± 0.97
Crude fat	91.6 ^b^ ± 1.01	94.5 ^a^ ± 0.67
Nitrogen free extract	88.1 ± 0.66	88.9 ± 0.93

^a,b^ Means in a row with different superscripts differ significantly (*p* < 0.05).

**Table 7:** Fecal characteristics of dogs fed the experimental diets (mean ± SD).

Parameter	PM-Based Diet	BSFL Meal-Based Diet
Defecation frequency/d	2.07 ± 0.64	2.40 ± 0.77
Score feces consistency	2.71 ^a^ ± 0.58	2.25 ^b^ ± 0.38
Score feces shaping	2.71 ^a^ ± 0.58	2.25 ^b^ ± 0.38
Fecal output (wet), g/5 d	492 ^b^ ± 34.0	569 ^a^ ± 37.8
Fecal output (DM), g/5 d	160 ± 14.1	158 ± 9.40
DM content (%)	33.0 ^a^ ± 2.62	28.0 ^b^ ± 2.50

^a,b^ Means in a row with different superscripts differ significantly (*p* < 0.05).

## Data Availability

The data presented in this study are available in this manuscript.
